# Efficacy of prosultiamine treatment in patients with human T lymphotropic virus type I-associated myelopathy/tropical spastic paraparesis: results from an open-label clinical trial

**DOI:** 10.1186/1741-7015-11-182

**Published:** 2013-08-15

**Authors:** Tatsufumi Nakamura, Tomohiro Matsuo, Taku Fukuda, Shinji Yamato, Kentaro Yamaguchi, Ikuo Kinoshita, Toshio Matsuzaki, Yoshihiro Nishiura, Kunihiko Nagasato, Tomoko Narita-Masuda, Hideki Nakamura, Katsuya Satoh, Hitoshi Sasaki, Hideki Sakai, Atsushi Kawakami

**Affiliations:** 1 Department of Molecular Microbiology and Immunology, Graduate School of Biomedical Sciences, Nagasaki University, 1-12-4 Sakamoto, Nagasaki 852-8523, Japan; 2 Department of Nephro-Urology, Graduate School of Biomedical Sciences, Nagasaki University, Nagasaki, Japan; 3 Translational Medicine Unit, Department of Clinical Neuroscience and Neurology, Graduate School of Biomedical Sciences, Nagasaki University, Nagasaki, Japan; 4 Department of Hospital Pharmacy, Nagasaki University Hospital, Nagasaki, Japan; 5 Neurology Section, Japanese Red Cross Nagasaki Genbaku Hospital, Nagasaki, Japan; 6 Department of Neurology, Okatsu Hospital, Kagoshima, Japan; 7 Neurology Section, Isahaya Health Insurance General Hospital, Nagasaki, Japan

**Keywords:** HAM/TSP, HTLV-I, Prosultiamine, Treatment

## Abstract

**Background:**

Human T lymphotropic virus type I (HTLV-I)-associated myelopathy/tropical spastic paraparesis (HAM/TSP) is a chronic myelopathy characterized by motor dysfunction of the lower extremities and urinary disturbance. Immunomodulatory treatments are the main strategy for HAM/TSP, but several issues are associated with long-term treatment. We conducted a clinical trial with prosultiamine (which has apoptotic activity against HTLV-I-infected cells) as a novel therapy in HAM/TSP patients.

**Methods:**

We enrolled 24 HAM/TSP patients in this open-label clinical trial. Prosultiamine (300 mg, orally) was administered once daily for 12 weeks. We monitored changes in the motor function of the lower extremities and urinary function as well as copy numbers of the HTLV-I provirus in peripheral blood mononuclear cells (PBMCs).

**Results:**

Improvement in the motor function of the lower extremities based on a reduction in spasticity (for example, decrease in time required for walking and descending a flight of stairs) was observed. In an urodynamic study (UDS), bladder capacity and detrusor pressure and then maximum flow rate increased significantly. Detrusor overactivity and detrusor-sphincter dyssynergia improved in 68.8% and 45.5% of patients observed at pretreatment, respectively. Improvement in UDS corresponded with improvements in the score of nocturia-quality of life questionnaire. HTLV-I proviral copy numbers in PBMCs decreased significantly (approximately 15.4%) compared with pretreatment levels.

**Conclusions:**

These data suggest that prosultiamine can safely improve motor dysfunction of the lower extremities and urinary disturbance as well as reduce HTLV-I provirus levels in peripheral blood. It therefore has potential as a new therapeutic tool for HAM/TSP patients.

**Trial registration:**

University Hospital Medical Information Network Clinical Trials Registry (UMIN-CTR) number, UMIN000005969.

Please see related commentary: http://www.biomedcentral.com/1741-7015/11/183.

## Background

Human T lymphotropic virus type I (HTLV-I) infects approximately 10 to 20 million people worldwide, mainly in large endemic areas such as southern Japan, the Caribbean, Central and South America, the Middle East, Melanesia, and equatorial regions of Africa [1,2]. HTLV-I is a human retrovirus and the causative agent of adult Tcell leukemia and HTLV-I-associated myelopathy/tropical spastic paraparesis (HAM/TSP) [3,4]. HAM/TSP is a chronic progressive myelopathy characterized by bilateral pyramidal tracts involved with sphincteric disturbances [5]. Only a small proportion of HTLV-I-infected individuals develop HAM/TSP. However, the main neurological symptoms (for example, motor dysfunction of the lower extremities accompanied by urinary disturbance) are progressive and lead to a deterioration in the quality of life (QoL) of patients once the myelopathy develops. Therefore, novel and safe therapeutic regimens are needed for HAM/TSP patients to use as treatment, or to prevent disease progression.

The primary neuropathological feature of HAM/TSP is chronic inflammation in the spinal cord caused by high HTLV-I proviral load in peripheral blood. Immunomodulatory therapy such as corticosteroid hormones and interferon (IFN)α has been the main treatment administered to HAM/TSP patients to date [6]. Although these treatments have produced good results in the short term, their overall efficacy is controversial [6,7]. In addition, it is not known if these treatments can be tolerated as a long-term or lifelong treatment against HAM/TSP, or whether they are necessary in the therapeutic strategy against HAM/TSP. When treating HAM/TSP, the optimal treatment is elimination of HTLV-I-infected cells from peripheral blood because HTLV-I-infected CD4^+^ T cells are the first responders in the immunopathogenesis of HAM/TSP [8].

(*N*-[(4-amino-2-methyl-5-pyrimidinyl) methyl]-*N*-[4-hydoxy-1-methyl-2-(propyldithio)-1-butenyl]-formamide) is known as prosultiamine and as Alinamin®. It is a product of Takeda Pharmaceutical Company Limited (Osaka, Japan). Prosultiamine is a homolog of allithiamine, which was originally synthesized from thiol-type vitamin B1 and allicin [9]. For stability in the blood and efficient access of vitamin B1 to tissues, prosultiamine was developed after allyl disulfide derived from allicin was substituted with propyl disulfide in the structure of allithiamine [10]. Importantly, prosultiamine is pharmacologically stable and is readily available for the treatment of Wernicke’s encephalopathy and polyneuropathy induced by deficiency of vitamin B1. Moreover, it has been shown to be safe for use in Japan. Therefore, this drug could be utilized immediately for conducting clinical trials in individuals with HAM/TSP.

Recently, we demonstrated that prosultiamine can induce the caspase-dependent apoptosis of HTLV-I-infected cells through disruption of intracellular redox reactions by a disulfide moiety in its structure [11]. Based on these data, we undertook a clinical trial based on the intravenous administration of prosultiamine in HAM/TSP patients with the purpose of targeting HTLV-I-infected cells [11]. We found that prosultiamine administration for 2 weeks was safe and induced clinical improvement. Examples of such improvement included a decrease in (i) spasticity of the lower extremities and (ii) levels of HTLV-I provirus in peripheral blood mononuclear cells (PBMCs) to 30% to 50% of pretreatment levels.

As mentioned above, we do not know if prosultiamine can be tolerated as a long-term treatment against HAM/TSP or indeed if it is necessary in the therapeutic strategy against HAM/TSP. Therefore, in the present study, we administered prosultiamine *via* the oral route for 12 weeks in subjects with HAM/TSP. We found that such treatment could result in (i) improved motor function in the lower extremities based on a decrease in spasticity, (ii) appreciable amelioration of associated urinary disturbance, and (iii) a decrease in the level of HTLV-I provirus in peripheral blood.

## Methods

### Ethical approval of the study protocol

This study protocol was approved by the clinical studies review boards of Nagasaki University Hospital (Nagasaki, Japan). The clinical trial was registered in the University Hospital Medical Information Network Clinical Trials Registry (UMIN-CTR) UMIN000005969. Written informed consent was obtained from all patients enrolled in the study for both participation in the study, and for inclusion of personal data as shown in Table 1.

**Table 1:** Profile of HAM/TSP patients enrolled and improvement of motor function in the lower extremities 12 weeks after treatment

	**Age (years)**	**Sex**	**Duration of illuness (years)**	**Concomitant therapy**			**Intermittent self-catheterization**	**OMDS**^**a**^		**Spasticity of the lower extremities**	
**Case no.**				**Immunomodulator**	**Drug for neurogenic bladder**						
					**Anticholinergic**	**α1 blocker**		**Before treatment**	**After treatment**	**Before treatment**	**Improvement**^**b**^
1	80	Female	23	No	Yes		Yes	6	6	Yes	Yes
2	64	Female	16	No	Yes		Yes	6	6	Yes	Yes
3	57	Male	51	PSL/ IFN-α	Yes		Yes	6	6	Yes	Yes
4	51	Female	36	No	Yes		Yes	9	9	Yes	Yes
5	67	Female	3	No			Yes	3	3	No	
6	61	Female	30	No			Yes	5	5	Yes	Yes
7	68	Female	12	No		Yes	No	4	4	Yes	Yes
8	64	Male	11	PSL		Yes	No	5	5	Yes	No
9	66	Male	23	PSL	Yes		Yes	9	9	Yes	Yes
10	76	Male	23	No			No	6	6	Yes	Yes
11	53	Female	7	No			Yes	6	6	Yes	No
12	62	Female	12	PSL		Yes	Yes	4	4	No	
13	44	Female	22	No			Yes	6	6	Yes	No
14	56	Male	10	No	Yes		Yes	5	5	Yes	Yes
15	71	Female	45	No	Yes		Yes	9	9	No	
16	78	Female	18	No	Yes		Yes	5	5	Yes	Yes
17	50	Female	19	No			No	5	5	Yes	Yes
18	63	Female	29	No	Yes		Yes	8	8	Yes	Yes
19	62	Female	9	PSL	Yes		Yes	8	8	Yes	No
20	60	Female	34	No	Yes		Yes	2	1	No	
21	46	Female	26	PSL		Yes	No	2	1	Yes	Yes
22	31	Female	7	No		Yes	No	4	3	Yes	Yes
23	56	Male	18	No			Yes	10	10	No	
24	56	Male	16	IFN-α			No	2	2	Yes	Yes
Remarks	mean ± SD;		mean ± SD;								% improvement:
	60.1 ± 11.2		20.9 ± 12.1								78.9 (P = 0.0003)^c^

^a^Osame’s motor function score (OMDS) was rated from 0 to 13 according to the disability grade [13].

^b^Improvement in spasticity of more than 1 grade according to the modifiedAshworth scale [14].

^c^Statistical significance was determined by the McNemar test.

*IFNα* interferon α, *PSL* prednisolone.

### Patients

We enrolled 24 HAM/TSP patients (17 women and 7 men; 31 to 80 years (mean ± SD; 60.1 ± 11.2 years)) who fulfilled criteria described previously [12]. The duration of illness was 3 to 51 years (mean ± SD; 20.9 ± 12.1 years). Motor function scores were rated from 0 to 13 according to the motor disability score described by Osame *et al*. [13]. Concomitant therapies such as immunomodulators and drugs for the neurogenic bladder were continued on the condition that the dose was kept constant during the study period. Intermittent self-catheterization with regard to voiding was noted except in cases 7, 8, 10, 17, 21, 22, and 24. Patient profiles are shown in Tables 1 and 2.

**Table 2:** Improvement of motor function in the lower extremities and urinary function 12 weeks after treatment

	**Time required to walk 10 m (sec)**			**Time required to walk downstairs (sec)**			**Detrusor overactivity**		**Detrusor-sphincter dyssnergia**	
**Case no.**	**Before treatment**	**After treatment**	**% improvement**	**Before treatment**	**After treatment**	**% Improvement**	**Before treatment**	**After treatment**	**Before treatment**	**After treatment**
1	26.5	21.6	18.5	N.E.			No	No	No	No
2	15.5	9.8	36.8	N.E.			Yes	No	No	No
3	11.5	10.5	8.7	8.6	7.7	10.5	Yes	No	Yes	Yes
4	N.E.			N.E.			Yes	No	Yes	Yes
5	5.3	4.9	7.5	3.8	3.7	2.6	No	No	Yes	No
6	5.9	6.2	-5.1	4.1	4.2	-2.4	No	No	Yes	No
7	8.9	9.5	-6.7	9.2	7.9	14.1	No	No	No	No
8	12.6	13.3	-5.6	9.5	8.6	9.5	Yes	No	Yes	Yes
9	N.E.			N.E.			Yes	No	No	No
10	20	25.1	-25.5	N.E.			No	No	No	No
11	29.5	32.5	-10.2	N.E.			Yes	No	Yes	Yes
12	6.6	6.9	-4.5	4.4	4.3	2.3	Yes	No	Yes	No
13	22.8	21.3	6.6	N.E.			Yes	No	No	No
14	15.4	11.3	26.6	14.3	11.4	20.3	No	No	No	No
15	N.E.	N.E.		N.E.			Yes	No	No	No
16	13.7	20.9	-52.6	N.E.			Yes	Yes	Yes	Yes
17	13.3	11.5	13.5	10.0	7.3	27	Yes	Yes	No	No
18	N.E.			N.E.			Yes	Yes	No	No
19	N.E.			N.E.			Yes	Yes	Yes	No
20	6.9	5.7	17.4	4.5	3.5	22.2	No	No	No	No
21	5.7	4.1	28.1	3.6	3.5	2.8	Yes	No	No	No
22	10.3	6.8	34	9.4	4.4	53.2	Yes	Yes	Yes	Yes
23	N.E.			N.E.			No	No	Yes	No
24	4.5	4.3	4.4	3.2	3.4	-6.3	Yes	No	No	No
Remarks								% improvement:		% improvement:
								68.8 (P = 0.0094)^a^		45.5 (P = 0.0736)^a^

^a^Statistical significance was determined by the McNemar test. N.E.; not evaluated because of high OMDS.

### Study design

#### ***Treatment protocol***

Prosultiamine was imported from Ildon Pharmaceutical Co., Ltd (Seoul, South Korea). Capsulated prosultiamine (300 mg, orally) was administered once daily for 12 weeks.

### Assessment of effect

#### ***Neurological assessment***

We monitored changes in neurological signs, motor disability scores, time required for walking 10 m, and time required for walking down a flight of stairs at 4-week intervals. Spasticity of the lower extremities was graded using the modified Ashworth scale (MAS) [14].

#### ***Urological assessment***

Subjective symptoms were evaluated using the scores of the Nocturia Quality of Life (N-QoL) questionnaire [15-17] at 4-week intervals. The N-QoL questionnaire comprised 13 items and dealt with daytime energy, worry, productivity, sleep, and vitality. The total score ranged from 0 (poorest QoL) to 100 (best QoL). The Duet® Logic G2 system (Mediwatch UK Ltd., Rugby, UK) was used for the urodynamic study (UDS). Bladder capacity, detrusor pressure, maximum flow rate, detrusor overactivity (DO) and detrusor-sphincter dyssynergia (DSD) were evaluated by UDS.

#### ***Quantification of HTLV-I proviral load***

For quantitative analyses of HTLV-I proviral loads, real-time quantitative polymerase chain reaction (RT-qPCR) was carried out in a Light-Cycler® FastStart DNA Master (Roche Diagnostics, Mannheim, Germany) based on fluorescence detection with SYBER® Green, as described previously [11]. Briefly, genomic DNA samples from PBMCs from HAM/TSP patients were prepared using a Genomic DNA Extraction kit (Wako Pure Chemical Industries, Ltd., Osaka, Japan) and were subjected to RT-PCR in a LightCycler PCR system using *Tax*-specific primers, that is, forward primer (5′- AAACAGCCCTGCAGATACAAAGT-3′) and reverse primer (5′-ACTGTAGAGCTGAGCCGATAACG-3′), as well as β-actin-specific primers, that is, forward primer (5′-GCCCTCATTTCCCTCTCA-3′) and reverse primer (5′-GCTCAGGCAGGAAAGACAC-3′). The PCR condition for *Tax* was 40 cycles of denaturation (95°C, 15 s), annealing (55°C, 5 s), extension (72°C, 10 s). That for β-actin was 32 cycles of denaturation (95°C, 15 s), annealing (62°C, 5 s), and extension (72°C, 15 s). The HTLV-I proviral load per 10,000 cells was calculated according to the following formula: (copy number of *Tax*)/(copy number of β-actin/2) × 10,000

### Statistical analyses

The Wilcoxon signed-rank test was used for statistical analyses of the change of HTLV-I proviral copy numbers and on the N-QoL scores or the urodynamic study except for DO and DSD. The McNemar test was used for statistical analyses of improvement of spasticity, DO and DSD. JMP 10 (SAS Institute Inc., Cary, NC, USA) was used as the software for statistical analyses. *P*< 0.05 was considered significant.

## Results

### Improvement of motor function of the lower extremities

Improvement in Osame’s motor function score (OMDS) was observed in three patients during treatment (Table 1). After 12 weeks of treatment, improvement of more than 1 grade of the degree of spasticity (evaluated according to MAS) was observed in 15 of 19 patients in whom spasticity of the lower extremities was observed before treatment (% improvement; 78.9, *P* = 0.0003, McNemar test) (Table 1). In time required for walking 10 m in 18 ambulatory patients, the decrease ranged from 4.4% to 36.8% was observed in 11 patients although the increase ranged from 4.5% to 52.6% was observed in 7 patients (Table 2). In time required for walking down a flight of stairs in 12 patients, the decrease ranged from 2.3% to 53.2% was observed in 10 patients although the increase of 2.4% or 6.3% was observed in 2 patients (Table 2).

### Improvement in urinary function

**Figure 1:**
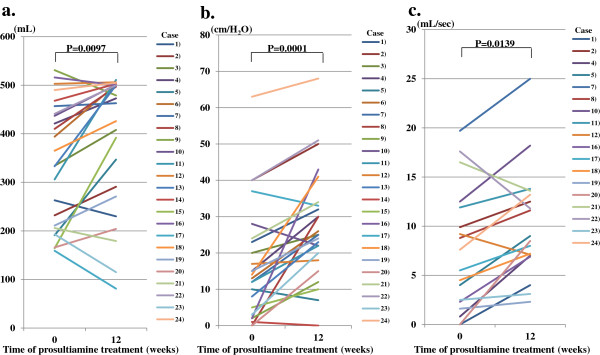
**Change in bladder function as evaluated by urodynamic study.** Bladder capacity **(a)** and detrusor pressure **(b)** increased significantly 12 weeks after prosultiamine treatment. Maximum flow rate **(c)** increased significantly 12 weeks after prosultiamine treatment as assessed in 18 patients whose voiding function alone was partially reserved. Statistical significance was determined by the Wilcoxon signed-rank test.

The conserved overall score of the N-QoL questionnaire was significantly improved, with a significant improvement of subscale scores at 12 weeks post treatment (Table 3). We compared urinary function by UDS at pretreatment with that at 12 weeks after treatment initiation. Bladder capacity and detrusor pressure were significantly increased from 341.3 (SD 127.2) ml to 391.0 (SD 139.9) ml (*P* = 0.0097), and 16.8 (SD 15.6) cm/H_2_O to 27.5 (SD 15.3) cm/H_2_O (*P* = 0.0001), respectively, by this treatment (Figure 1a,b). As analyzed in 18 patients whose own voiding function was partially reserved, the maximum flow rate was increased significantly from 7.5 (SD 6.2) ml/s to 10.2 (SD 5.6) ml/s (*P* = 0.0139) (Figure 1c). Moreover, DO improved in 68.8% (11 of 16 patients observed at pretreatment) by this treatment (*P* = 0.0094, McNemar test) (Table 2). DSD also improved in 45.5% (5 of 11 patients observed at pretreatment) 12 weeks after the start of treatment (*P* = 0.0736, McNemar test) (Table 2).

**Table 3:** Changes in N-QoL scores after 12 weeks treatment with prosultiamine

**Question**	**Before treatment**	**After treatment**	***P*** **value**
Q1 Concentration	0.6 ± 1.0	0.3 ± 1.6	0.1235
Q2 Low in energy	0.9 ± 1.1	0.4 ± 0.6	0.0077
Q3 Sleep during the day	1.5 ± 1.4	1.0 ± 1.1	0.0229
Q4 Productiveness	0.7 ± 0.9	0.3 ± 0.6	0.0830
Q5 Physical activities	1.0 ± 1.2	0.5 ± 0.8	0.0505
Q6 Fluid restriction	0.8 ± 1.2	0.7 ± 0.9	0.3270
Q7 Inadequate sleep at night	1.6 ± 1.5	0.7 ± 1.0	0.0070
Q8 Disturbance of others	0.8 ± 1.9	0.5 ± 1.8	0.0277
Q9 Preoccupation with waking at night	0.6 ± 1.1	0.3 ± 0.6	0.1235
Q10 Worry over condition worsening	1.5 ± 1.5	0.8 ± 1.1	0.0032
Q11 Worried over treatment options	1.5 ± 1.6	1.0 ± 1.3	0.0303
Q12 Overall bother	1.3 ± 1.3	0.8 ± 0.8	0.0238
Q13 Overall impact on everyday life	2.6 ± 2.8	0.9 ± 1.0	0.0023
Converted overall score (Q1 to 12)	73.2 ± 21.0	85.3 ± 19.9	0.0001
Subscale scores:			
Sleep/Energy (Q1 to5, 7)	74.0 ± 20.7	87.0 ± 15.0	0.0001
Bother/Concern (Q6, Q8 to12)	72.4 ± 25.7	83.7 ± 21.2	0.0028

Subjective symptoms were evaluated using the scores of the Nocturia Quality of Life (N-QoL) questionnaire [15-17]. The score ranges of each question except Q13 are from 0 to 4. The score of Q13 is from 0 to 10. Converted overall score (0–100) = 100 × total converted scores (Q1 to 12)/4 × question numbers, converted score = 4-each raw score. Subscale score (0 to 10) = 100 × total converted scores/4 × question numbers. Statistical significance was determined by the Wilcoxon signed-rank test.

### Decrease in HTLV-I proviral copy numbers in PBMCs

**Figure 2:**
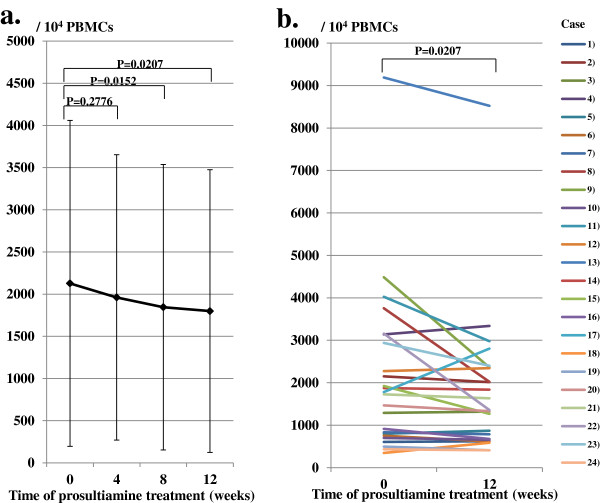
**Change in human T lymphotropic virus type I (HTLV-I) proviral copy numbers in peripheral blood mononuclear cells (PBMCs). (a)** HTLV-I proviral copy numbers from 10^4^ PBMCs decreased gradually until 12 weeks after prosultiamine treatment. The level of HTLV-I proviral copy numbers 12 weeks after prosultiamine treatment decreased by 15.4% compared with the time at pretreatment. **(b)** Changes in HTLV-I proviral copy numbers in each case between pretreatment and 12 weeks after prosultiamine treatment. Statistical significance was determined by the Wilcoxon signed-rank test.

We monitored changes in copy numbers of the HTLV-I provirus in PBMCs at pretreatment as well as 4, 8, and 12 weeks after treatment commencement (Figure 2a). HTLV-I proviral copy numbers in 10^4^ PBMCs decreased gradually from 2,127 (SD 1,932) at pretreatment to 1,961 (SD 1,692) (*P* = 0.2776, vs pretreatment), 1,845 (SD 1,693) (*P* = 0.0152, vs pretreatment) and 1,799 (SD 1,676) (*P* = 0.0207, vs pretreatment) at 4, 8, and 12 weeks after treatment, respectively. The level of HTLV-I proviral copy numbers 12 weeks after treatment decreased significantly (approximately 15.4%) from pretreatment levels. Figure 2b shows the changes in HTLV-I proviral copy numbers in each case between pretreatment and 12 weeks after treatment commencement. A decrease of approximately 30% to 50% in HTLV-I proviral copy numbers was observed in cased 8, 9, 11, 15 and 22.

### Adverse effects

There were no serious adverse effects except mild epigastric discomfort rated as ‘2’ evaluated according to the Global Overall Symptom scale [18] in three HAM/TSP patients. This symptom immediately resolved after this clinical trial.

## Discussion

Effective therapeutic regimens are needed urgently to treat such myelopathic symptoms of HAM/TSP as spasticity of lower extremities and urinary disturbance. To this end, we administered prosultiamine *via* the oral route for 12 weeks in subjects with HAM/TSP. This treatment improved (i) the motor ability of the lower extremities by decreasing spasticity, and (ii) urinary function. The mean duration of illness of the patients enrolled in this study was relatively long (approximately 21 years), so the efficacy of this treatment is promising. Indeed, these data suggest that the pathological processes in the spinal cord of HAM/TSP patients are partially reversible and treatable even if the tissues are damaged over a long period of time.

The most striking effect in this clinical trial was the amelioration of urinary disturbance in HAM/TSP patients. The common urodynamic findings in HAM/TSP patients are DO, DSD and detrusor hypoactivity [19]. However, as evaluated by UDS, prosultiamine treatment resulted in a significant increase in detrusor pressure and bladder capacity followed by an increase in maximum flow rate with improved DO. DSD also improved in 45.5% (5 of 11 patients observed at pretreatment) (*P* = 0.0736). Although this value did not reach statistical significance, it showed a tendency of improvement. This is the first time that the therapeutic effect for urinary dysfunction in HAM/TSP patients was evaluated in detail by UDS. With respect to the effect of urinary conditions on the QoL of HAM/TSP patients, nocturia, urgency, increased frequency of urination and dysuria have been reported to be the main problems [20]. Therefore, we evaluated the change in QoL of patients using N-QoL questionnaires during treatment. The improved UDS corresponded with improvements in the score of N-QoL questionnaires. Concomitant pharmacological therapies for the neurogenic bladder were continued during the present study. However, the efficacy of prosultiamine treatment, even in patients who were not having concomitant therapies (cases 5, 6, 11, 13, 23, and 24), strongly suggested that urological improvement was dependent solely upon prosultiamine treatment (Table 1). Overall, these data suggest that prosultiamine treatment can reverse bladder dysfunction in HAM/TSP patients.

Recently, two reports have focused on targeting HTLV-I in therapeutic trials against HAM/TSP. One study used reverse transcriptase (RT) inhibitors, whereas the other used a histone deacetylase enzyme inhibitor for treatment [21,22]. In the former, the results of combination therapy (zidovudine + lamivudine) in a randomized, double-blind, placebo-controlled study suggested that RT inhibitors were not effective for targeting HTLV-I for the treatment of HAM/TSP. In the latter study, long-term treatment using valproic acid did not reduce the number of HTLV-I-infected cells in peripheral blood [22]. A decrease in the HTLV-I provirus in PBMCs was one of the primary endpoints in our recent report [11]. Indeed, oral administration of prosultiamine induced a significant decrease in HTLV-I proviral copy numbers in PBMCs, However, the rate of reduction was not as high as we had expected. This finding might suggest a limitation of the protocol used in the present study. Thus, the remarkable improvement of motor dysfunction and urinary function in the present study cannot be attributed solely to a decrease in HTLV-I proviral copy numbers in PBMCs. The exact mechanism is not known. Prosultiamine was originally developed for efficient access of vitamin B1 to nervous tissues [10]. Although this drug is reduced to a part thiamine and propyl disulfide by the intracellular reducing system after penetration to the cells [10], it is suspected that the disruption of intracellular redox system is induced during reduction of disulfide bond leading to the apoptosis of HTLV-I-infected cells [11]. Therefore, it might be conceivable that, as one of the mechanisms, this drug functions to induce the apoptosis of HTLV-I-infected cells in the spinal cord even if the extent of reduction of the number of HTLV-I-infected cells in PBMCs is relatively small. Further investigations including analysis of cerebrospinal fluid are needed to elucidate the exact mechanism of action of prosultiamine.

## Conclusions

In the present work we have demonstrated that oral administration of prosultiamine can safely promote improvement of motor function of the lower extremities based on a reduction of spasticity along with appreciable amelioration of urinary disturbance associated with a decrease in the amount of HTLV-I provirus in peripheral blood. Our results suggest that prosultiamine could be a promising therapeutic tool for HAM/TSP patients. Therefore, further studies are warranted, such as the evaluation of prosultiamine treatment against HAM/TSP in a large-scale, randomized, controlled study.

## Competing interests

The authors declare that they have no competing interests.

## Authors’ contributions

TN designed the study, assessed the neurological findings, analyzed data, and wrote the paper. TMatsuo designed the study, analyzed data, and contributed to the urological studies. HSakai contributed to the urological studies. TF and TN-M assessed the neurological findings. TN, TF and SY managed the blood supply and laboratory studies. KY and HSasaki handled the prosultiamine and enclosed it in capsules. IK, TMatsuzaki, YN, KN, HN, KS, and AK were involved in managing the patients. All authors contributed to the manuscript and approved the final version of the report.
